# Polymorphisms in *VDR*, *CYP27B1*, *CYP2R1*, *GC* and *CYP24A1* Genes as Biomarkers of Survival in Non-Small Cell Lung Cancer: A Systematic Review

**DOI:** 10.3390/nu15061525

**Published:** 2023-03-21

**Authors:** Laura Elena Pineda-Lancheros, José María Gálvez-Navas, Susana Rojo-Tolosa, Cristina Membrive-Jiménez, María Isabel Valverde-Merino, Fernando Martínez-Martínez, Almudena Sánchez-Martín, MCarmen Ramírez-Tortosa, Cristina Pérez-Ramírez, Alberto Jiménez-Morales

**Affiliations:** 1 Pharmacogenetics Unit, Pharmacy Service, University Hospital Virgen de las Nieves, Avda. de las Fuerzas Armadas 2, 18004 Granada, Spain; 2 Department of Biochemistry and Molecular Biology II, Faculty of Pharmacy, Campus Universitario de Cartuja, Universidad de Granada, 18011 Granada, Spain; 3 Cancer Registry of Granada, Andalusian School of Public Health, Carretera del Observatorio, 4, 18011 Granada, Spain; 4 Respiratory Medicine Department, University Hospital Virgen de las Nieves, 18014 Granada, Spain; 5 Pharmaceutical Care Research Group, Faculty of Pharmacy, University of Granada, 18071 Granada, Spain; 6 Department of Pharmacy and Pharmaceutical Technology, Social and Legal Assistance Pharmacy Section, Faculty of Pharmacy, University of Granada, 18071 Granada, Spain

**Keywords:** non-small cell lung cancer, vitamin D, overall survival, progression-free survival, single-nucleotide polymorphisms, *VDR*, *CYP27B1*, *CYP2R1*, *GC*, *CYP24A1*

## Abstract

The objective of this systematic review was to provide a compilation of all the literature available on the association between single-nucleotide polymorphisms (SNPs) in the genes involved in the metabolic pathway of vitamin D and overall survival (OS) and progression-free survival (PFS) in patients with non-small cell lung cancer (NSCLC). This systematic review was conducted in accordance with the PRISMA guidelines. It included all the literature published up to 1 November 2022 and was carried out in four databases (Medline [PubMed], Scopus, Web of Science, and Embase), using the PICO strategy, with relevant keywords related to the objective. The quality of the studies included was evaluated with an assessment tool derived from the Strengthening the Reporting of Genetic Association Studies (STREGA) statement. Six studies were included in this systematic review. Our findings showed that the BsmI (rs1544410), Cdx-2 (rs11568820), FokI (rs2228570), ApaI (rs7975232), TaqI (rs731236), rs4646536, rs6068816, rs7041, and rs10741657 SNPs in the genes that play a part in vitamin D synthesis (*CYP2R1*, *CYP27B1*), transport (*GC*), and metabolism (*CYP24A1*), as well as in the vitamin D receptor (*VDR*), are associated with OS and/or PFS in patients with NSCLC. The SNPs in *VDR* have been the most extensively analyzed. This systematic review summed up the available evidence concerning the association between 13 SNPs in the main genes involved in the vitamin D metabolic pathway and prognosis in NSCLC. It revealed that SNPs in the *VDR*, *CYP27B1*, *CYP24A1*, *GC*, and *CYP2R1* genes could have an impact on survival in this disease. These findings suggest the identification of prognostic biomarkers in NSCLC patients. However, evidence remains sparse for each of the polymorphisms examined, so these findings should be treated with caution.

## 1. Introduction

According to the latest data published by the World Health Organization (WHO), cancer is a leading cause of death worldwide, accounting for approximately six out of every ten deaths. The most common cancers are breast, lung, colon and rectum, and prostate [1]. In the world population, considering both sexes, lung cancer has the highest mortality (18%) and the second highest incidence (11.4%) [2]. The latest cancer statistics in the United States (2022) estimate that more than 350 people will die each day from lung cancer, which is more than breast, prostate, and pancreatic cancers combined and 2.5 times more than colorectal cancer, the second leading cause of cancer death [3]. 

Carcinogenesis can be triggered by a wide range of exogenous and endogenous factors, such as chemical exposure and genetic mutations. Molecular and cellular signaling in carcinogenesis begins with mutations in critical genes (e.g., MYC, RAS, ERK, TP53, KRAS), which drives aberrant cell growth, development, proliferation, apoptosis, differentiation, and migration [4]. The lung has various types of cells, most of which are epithelial cells. According to the size of the tumor cells, this type of neoplasm is classified as small cell lung cancer or non-small cell lung cancer (NSCLC), which accounts for 82% of cases [5,6]. NSCLC is characterized by affecting patients over the age of 65 [5]. There are determinants of survival in this type of carcinoma, including early diagnosis, the stage of the disease, and the treatment received [7,8,9]. Because of the high mortality rate in this kind of neoplasm, even after treatment with radiotherapy, chemotherapy, and/or surgery, there is a need to continue the search for new diagnostic and prognostic biomarkers for lung cancer, and here vitamin D stands out due to its functional versatility [10]. Its activity includes inhibition of cell proliferation in response to changes in the activity of the proteins that regulate the cell cycle, development of apoptosis, and cell differentiation, as well as antiangiogenic, antioxidant, and anti-inflammatory functions [11,12,13]. 

**Figure 1:**
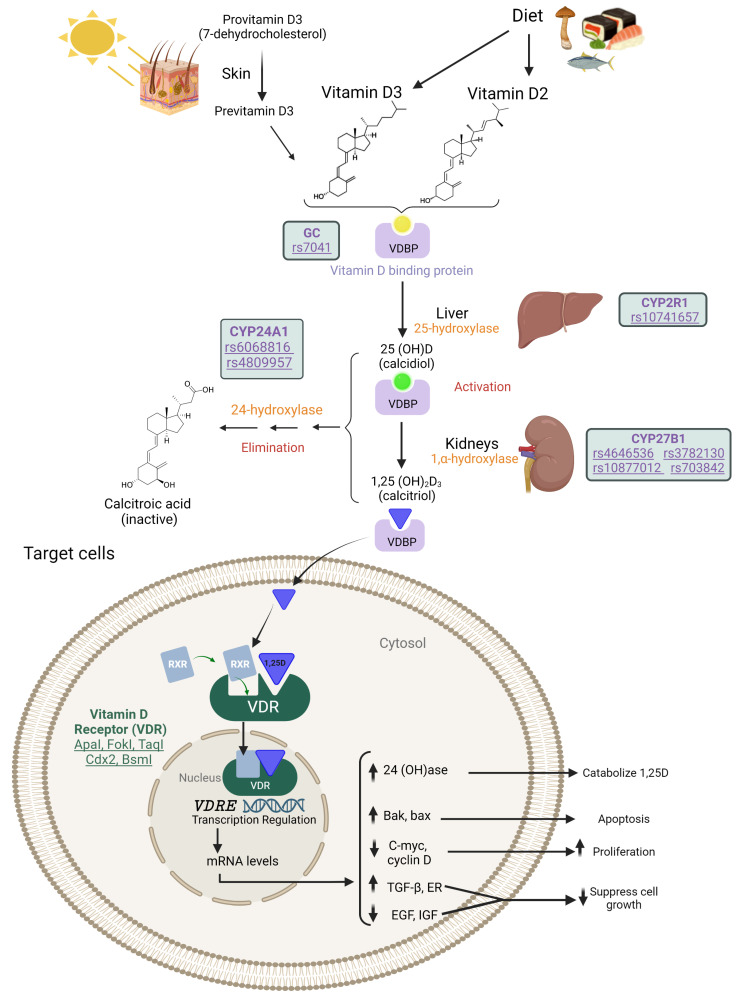
Metabolic pathway of vitamin D.

The term vitamin D encompasses several liposoluble molecules: ergocalciferol, cholecalciferol, calcidiol, and calcitriol. Cholecalciferol is synthesized in the dermis by the action of UVB radiation (290–320 nm) on 7-dehydrocholesterol, whereas ergocalciferol is supplied to the body through the diet [10,14,15]. Both vitamin D isoforms are transported in the blood bound to vitamin D binding protein (VDBP) [16]. To be activated, the isoforms need to undergo two hydroxylation reactions. The first occurs in the liver at position 25 through the action of the 25-hydroxylase or CYP2R1 enzyme, resulting in calcidiol or 25(OH)D. This is the metabolite that circulates for the longest in the blood and is therefore the one that is measured to determine serum vitamin D levels [14]. Then, a second hydroxylation takes place in the kidney through the action of the 1-alpha-hydroxylase or CYP27B1 enzyme, producing calcitriol or 1,25(OH)D. The latter is the active form of vitamin D, which binds to the vitamin D receptor (VDR) in the cell membrane [17,18,19]. Through this bond it is translocated to the nucleus, where the dimer binds to the nuclear receptor retinoid (RXR), acting as a transcription factor for vitamin D response elements (VDREs) [18,19]. Finally, calcitriol and calcidiol are inactivated by successive hydroxylation reactions through the action of the CYP24A1 enzyme; these increase the solubility of the molecule and enable it to be excreted renally (Figure 1) [20,21]. 

These VDREs enable the expression of a host of genes, which are related to key processes in the inception and development of cancer. This evidence suggests that low serum calcidiol levels or dysfunctionality of the vitamin may favor carcinogenesis, due to the presence of single-nucleotide polymorphisms (SNPs) in the genes that participate in vitamin D metabolism (*GC* or *VDBP*, *VDR*, *CYP2R1*, *CYP27B1*, and *CYP24A1*) [22,23,24]. There are studies that have evaluated the influence of SNP prevalence in these genes associated with risk, survival, progression-free survival, and response to platinum-based chemotherapy in lung cancer, specifically NSCLC [10,14,25,26,27,28,29,30,31,32,33]. 

Based on the above, there is a need for continued research toward discovering new biomarkers in NSCLC that facilitate screening and early diagnosis of the disease and make it possible to achieve higher survival rates. This study, therefore, aims to provide a compilation of the existing evidence on the role played in NSCLC survival by SNPs with a minor allele frequency greater than 1% in the *GC* (rs7041), *CYP2R1* (rs10741657), *CYP27B1* (rs10877012, rs4646536, rs3782130, and rs703842), *VDR* (BsmI [rs1544410], TaqI [rs731236], ApaI [rs7975232], FokI [rs2228570], Cdx2 [rs11568820]), and *CYP24A1* (rs6068816 and rs4809957) genes, according to the information in the dbSNP database [34,35,36].

## 2. Materials and Methods

### 2.1. Search Strategy

This systematic review was conducted in accordance with the PRISMA guidelines [37] (the PRISMA checklist is reported in Supplementary Table S1). It included all the studies published up to 1 November 2022 that evaluate the influence of any of the main SNPs involved in the vitamin D metabolic pathway on overall survival and/or progression-free survival in patients diagnosed with NSCLC. To identify eligible studies, we performed a computer search in the Medline (PubMed), Web of Science, Scopus, and Embase electronic databases up to the first week of November 2022. We looked for potentially relevant articles using the following search strategy, based on PICO methodology (Table 1) [38]:

No language or publication date restrictions were applied. The references in the articles selected were scanned to identify additional relevant articles.

### 2.2. Eligibility Criteria

#### 2.2.1. Inclusion Criteria

The inclusion criteria were as follows: (I) Studies conducted on humans, of any age and any population, who had been diagnosed with NSCLC. (II) Studies that evaluated SNPs in the *VDR*, *CYP27B1*, *CYP2R1*, *GC*, and *CYP24A1* genes, intermediaries in the vitamin D metabolic process. (III) Studies that reported quantitative results between the SNPs analyzed and the disease outcome. (IV) Studies that assessed general mortality and/or disease progression as outcomes (overall survival [OS]; progression-free survival [PFS]). (V) Cohort-type studies (retrospective and prospective), clinical trials, and randomized clinical trials. (VI) Studies published in any year, with full text availability. (VII) In the case of studies containing a cohort or part of a cohort that had been described previously, the most recent or extensive study was selected. Conversely, studies that presented the same cohort or part of a cohort but evaluated different genes of interest or different outcome measurements were included.

#### 2.2.2. Exclusion Criteria

The exclusion criteria were as follows: (I) Studies based solely on haplotypes. (II) Gene expression studies*. (III) Studies assessing vitamin D levels*. (IV) Studies assessing protein levels*. (V) Vitamin D supplementation studies*. (VI) Mixed cancer cohort studies that do not specifically report the site. (VII) Case report/case series-type articles, conference proceedings, editorials, letters to the editor, clinical guidelines, book chapters, and reviews.

* The studies that have been excluded did not analyze single nucleotide polymorphisms.

### 2.3. Data Collection and Analysis

#### 2.3.1. Study Selection

After deleting the duplicates, two researchers (L.E.P.L. and M.I.V.M.) independently screened the titles and abstracts. Disagreements were resolved through discussion with a third researcher (C.P.R). The full texts were retrieved and examined for eligible studies.

#### 2.3.2. Data Extraction

Data extraction from the articles included was performed by 2 authors independently (L.E.P.L. and S.R.T.). We developed a data extraction table that included the following information: basic characteristics of the study (first author, year of publication, and type of population [country]), description of the sample (total number of participants, number of deaths during study follow-up), and association of gene variants involved in vitamin D metabolism. In addition, the two reviewers extracted the data on measures of association in another table, which included associated SNPs (gene [location], rs number, nucleotide change [location], reported allele frequency [ALFA]) [36]; association of SNPs with OS and/or PFS, including the reference allele, log-rank *p* value, hazard ratio, and confidence interval (if available). Any discrepancy was resolved through discussion or by consulting a third researcher (A.S.M.). The terminology employed by each author was used to record the outcome measures.

### 2.4. Quality Assessment

To assess the quality of the studies included, a descriptive analysis was performed by means of a review by two researchers (L.E.P.L. and J.M.G.N.) of 9 items obtained from the Strengthening the Reporting of Genetic Association Studies (STREGA) statement [39]. In the event of any discrepancy, a third researcher (C.P.R.) was consulted. The items analyzed cover the following topics: (a) laboratory methods, (b) number of samples genotyped and genotyping success rate, (c) population stratification methodology, (d) genotype or haplotype inference methods, (e) Hardy–Weinberg equilibrium (HWE), and (f) indication of the novelty of the genetic association study. Scoring was as follows: if the study fully complied with item (Y), a value of 1 was assigned, if the item was incomplete (I), a value of 0.5 was assigned, and if the study did not comply (N), a value of 0 (zero) was assigned. A total score was calculated for each study by adding up the scores for the items evaluated (range 0 to 9). The score obtained was expressed as a percentage ([points obtained/maximum points] × 100). Finally, the percentage was taken as a reference to classify the studies as high (>80%), moderate (50–80%), or low (<50%) quality, as reported in previous studies [40,41,42].

## 3. Results

### 3.1. Search Results

**Figure 2:**
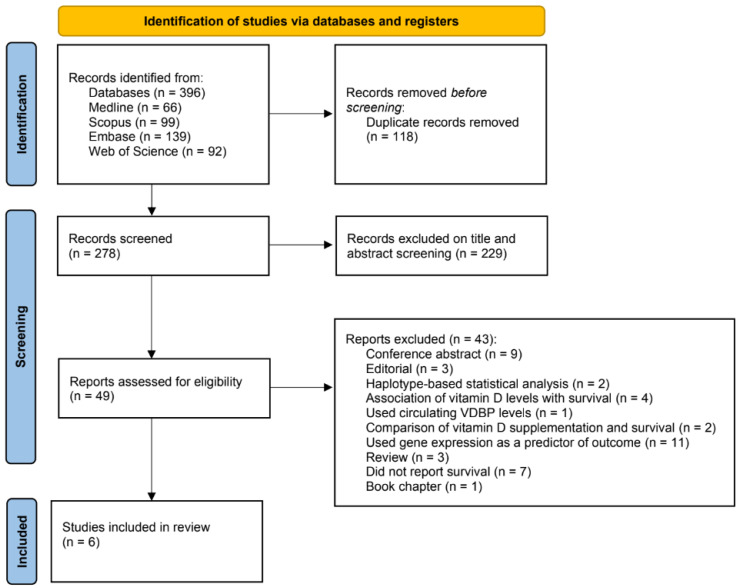
Systematic review study selection PRISMA flow diagram.

The initial search in the databases consulted produced a total of 396 articles (Figure 2). After the deletion of duplicates (n = 118) and articles that did not comply with the inclusion criteria in the screening of titles and abstracts (n = 229), 49 articles were preselected for full-text review. Of these, 43 were eliminated for the following reasons: 9 were conference proceedings, 3 were editorials, 2 performed the association analysis by haplotypes and not by single variants, 4 only analyzed the association between vitamin D levels and survival, 1 used only circulating levels of VDBP as a measure of association, 2 made the comparison between vitamin D supplementation and survival, 11 used gene expression as a survival prediction factor, 3 were reviews, 7 did not take survival as the outcome, and 1 was a book chapter. Finally, six articles were selected for this review.

### 3.2. Study Characteristics

The studies included were published between 2005 and 2021 [10,25,26,27,28,29]. All of them are cohort studies. Three were conducted on Asian populations (from China) [26,27,28], two in Caucasian populations (from the USA) [25,29], and one in a Caucasian population in southern Spain [10]. All the studies assessed patients diagnosed with NSCLC. One of them included only early-stage patients [29], two included only advanced-stage patients [25,28], and three included all stages (I–IV) [10,26,27]. Five of the six studies analyzed from three to five polymorphisms in the *VDR* gene [10,25,27,28,29] and two analyzed SNPs in the *CYP2R1*, *CYP27B1*, *GC*, and *CYP24A1* genes [10,26]. Only one study included all five genes, *CYP2R1*, *CYP27B1*, *GC*, *CYP24A1,* and *VDR*, in its analysis [10]. All the studies evaluated the influence of SNPs on OS. However, only three also assessed the influence of polymorphisms on PFS [10,28,29]. The main characteristics of the studies included are presented in Table 2.

### 3.3. Influence of Genetic Polymorphisms on Survival in NSCLC

The polymorphisms associated with OS and PFS in patients diagnosed with NSCLC are summarized in Table 3. 

#### 3.3.1. *VDR*: Vitamin D Receptor

In this review, information was collected on five SNPs located in the *VDR* gene: rs1544410 (BsmI), rs11568820 (Cdx-2), rs2228570 (FokI), rs7975232 (ApaI), and rs731236 (TaqI). Five studies included at least three of these in their analyses [10,25,27,28,29]. The rs1544410 (BsmI) polymorphism was included in five studies [10,25,27,28,29], of which two found statistically significant associations with regard to OS [10,27]. Liu et al. found in their multivariate Cox regression analysis that the CT and TT genotypes for the rs1544410 SNP were associated with a higher risk of death compared to the CC genotype in 562 patients of Asian origin (from China) with stage I–IV NSCLC (*p* = 0.008; HR = 1.55; 95% CI = 1.09–2.2; CT vs. CC, and *p* = 0.008; HR = 4.33; 95% CI = 1.34–14.0; TT vs. CC). Similarly, carriers of the rs1544410-T allele showed a higher risk of death compared to the CC genotype (*p* = 0.004; HR = 1.64; 95% CI = 1.16–2.31; T vs. CC) [27]. Furthermore, Pineda et al. found in the univariate Cox regression analysis that patients carrying the TT genotype had a higher risk of death than those with the C allele in 146 Caucasian patients (from Spain) with non-resected NSCLC (*p* = 0.0073; HR = 2.08; 95% CI = 1.22–3.56; TT vs. C) [10]. Progression-free survival was analyzed in three studies. However, no significant findings were obtained in any of them [10,28,29].

As for the rs11568820 (Cdx-2) polymorphism, two of the four studies that analyzed it found results concerning its association with OS and PFS [10,25,27,29]. Firstly, Zhou et al. made a sub-classification of their study population, and for 108 patients of Caucasian origin (from the United States) with early-stage NSCLC and with the histological type of squamous cell carcinoma, they found in the multivariate Cox regression model that those carrying the AG and AA genotypes showed a lower risk of death compared to the GG genotype (*p* = 0.05; HR = 0.55; 95% CI = 0.32–0.95; AG vs. GG, and *p* = 0.05; HR = 0.69; 95% CI = 0.16–2.96; AA vs. GG). Likewise, carriers of the A allele had a lower risk of death compared to the GG genotype (*p* = 0.04; HR = 0.56; 95% CI = 0.33–0.95; A vs. GG). Similar results were found when evaluating PFS, where carriers of the A allele showed a lower risk of progression than carriers of the GG genotype (*p* = 0.03; HR = 0.57; 95% CI = 0.34–0.94; A vs. GG) [29]. Secondly, Pineda et al. report that in a subgroup of 48 Caucasian patients (from Spain) with resected NSCLC, carriers of the rs11568820-AA genotype presented a higher risk of death than carriers of the G allele (*p* = 0.0129; HR = 7.43; 95% CI = 1.53–36.15; AA vs. G) in the multivariate Cox regression. This association maintained a strong trend for PFS in the univariate Cox regression model, where carriers of the AA genotype showed a tendency toward a higher risk of progression compared to carriers of the G allele (*p* = 0.055; HR = 4.34; 95% CI = 0.97–19.5; AA vs. G) [10].

The rs2228570 (FokI) polymorphism is studied in four of the articles included in this review [10,25,28,29]. However, only one study found a significant relationship between this polymorphism and OS. Heist et al. report that in 294 patients of Caucasian origin (from USA) with advanced NSCLC, in the multivariate Cox regression model, patients carrying the CT and TT genotypes showed a higher risk of death than those carrying the CC genotype (*p* = 0.04; HR = 1.32; 95% CI = 0.98–1.77; CT vs. CC, and *p* = 0.04; HR = 1.41; 95% CI = 0.96–2.07; TT vs. CC) [23]. The influence of FokI on PFS was assessed in three studies. However, none of them report significant findings [10,28,29].

Three of the studies included in this review investigated the influence of the rs7975232 (ApaI) SNP on survival [10,27,28]. Two of them found an association between this polymorphism and both OS and PFS [10,28]. Xiong et al., in a cohort of 755 Asian patients (from China) with advanced NSCLC, report in the multivariate Cox regression model that carriers of the AA genotype had a higher risk of death than carriers of the CC genotype (*p* < 0.001; HR = 2.84; 95% CI = 2.63–3.94; AA vs. CC). This association showed a strong trend in PFS, where the rs7975232-AA genotype displayed a tendency toward a higher risk of progression than the CC genotype (*p* = 0.053; HR = 1.43; 95% CI = 0.99–2.78; AA vs. CC) [28]. Similarly, in a later study with 146 Caucasian patients (from Spain) with non-resected NSCLC, in the univariate Cox regression model, carriers of the rs7975232-AA genotype showed a higher risk of death than carriers of the C allele (*p* = 0.0068; HR = 1.73; 95% CI = 1.16–2.58; AA vs. C). This association was clearer in PFS, wherein the multivariate Cox regression model carriers of the rs7975232-AA genotype presented a higher risk of progression compared to the C allele (*p* = 0.0002; HR = 3.08; 95% CI = 1.71–5.54; AA vs. C) [10]. 

The rs731236 (TaqI) SNP was investigated in three of the studies included in this review [10,27,28]. A significant association with OS and PFS was found in two of them [10,27]. First, Liu et al. assessed the influence of this SNP on OS and PFS in 586 Asian patients (from China) with NSCLC and found in the multivariate Cox regression model that those carrying the AG and GG genotypes showed a higher risk of death than those carrying the AA genotype (*p* = 0.027; HR = 1.41; 95% CI = 1.00–1.99; AG vs. AA, and *p* = 0.027; HR = 4.26; 95% CI = 1.32–13.8; GG vs. AA). Likewise, carriers of the rs731236-G allele had a higher risk of death than those with the AA genotype (*p* = 0.016; HR = 1.49; 95% CI = 1.07–2.08; G vs. AA) [27]. Another study of 146 Caucasian patients (from Spain) with non-resected NSCLC showed that in the multivariate Cox regression model, carriers of the rs731236-GG genotype exhibited a higher risk of death compared to carriers of the A allele (*p* = 0.0005; HR = 2.71; 95% CI = 1.55–4.75; GG vs. A). In addition, this association was maintained in the multivariate Cox regression model for PFS, where carriers of the rs731236-GG genotype showed a higher risk of progression than carriers of the A allele (*p* = 0.0463; HR = 1.74; 95% CI = 1.01–2.99; GG vs. A) [10]. 

#### 3.3.2. *CYP27B1*: Cytochrome P450 Family 27 Subfamily B Member 1

In this review, there were two studies that investigated the relationship of genetic polymorphisms in the *CYP27B1* gene to survival in NSCLC [10,26]. For the rs10877012 SNP, Pineda et al., in their analysis of the subgroup of 146 Caucasian patients (from Spain) with non-resected NSCLC, demonstrated in the univariate Cox regression model that patients carrying the GG genotype displayed a higher risk of progression than carriers of the T allele (*p* = 0.044; HR = 2.05; 95% CI = 1.02–4.14; GG vs. T). The association of this SNP was not observed in the analysis of its influence on OS [10]. Moreover, Kong et al. did not observe an association between rs10877012 and OS [26]. As for the rs4646536 polymorphism, a study with 194 Caucasian NSCLC patients (from Spain) reports that in the multivariate Cox regression model, carriers of the rs4646536-A allele showed a tendency toward association with OS compared to carriers of the GG genotype (*p* = 0.056; HR = 2.01; 95% CI = 0.98–4.14; A vs. GG). This observation was more evident in PFS, where carriers of the A allele had a higher risk of progression than those with the GG genotype (*p* = 0.023; HR = 2.11; 95% CI = 1.11–4.04; A vs. GG). In addition, in the subgroup analysis, it was found that in 146 patients with non-resected NSCLC, in the multivariate Cox regression model, carriers of the rs4646536-AA genotype presented a higher risk of progression compared to the G allele (*p* = 0.004; HR = 8.77; 95% CI = 1.94–39.7; AA vs. G). The rs3782130 SNP showed a significant association in the univariate Cox regression model with respect to PFS in 146 Caucasian patients (from Spain) with non-resected NSCLC. In particular, carriers of the GG genotype exhibited a higher risk of progression relative to carriers of the C allele (*p* = 0.045; HR = 2.05; 95% CI = 1.01–4.13; GG vs. C) [10]. This association was not observed in the influence of the rs3782130 polymorphism on OS [10,26]. The rs703842 genetic polymorphism was included in the study conducted by Kong et al. without significant findings with regard to OS (*p* = 0.627) [26].

#### 3.3.3. *CYP24A1*: Cytochrome P450 Family 24 Subfamily A Member 1

Some studies assessed the relationship between the rs6068816 and rs4809957 polymorphisms in the *CYP24A1* gene and survival in NSCLC [10,26]. With regard to the rs6068816 SNP, Pineda et al. report in the multivariate Cox regression model that in 146 Caucasian patients (from Spain) with non-resected NSCLC, those carrying the TT genotype showed a higher risk of death than carriers of the C allele (*p* = 0.0089; HR = 3.47; 95% CI = 1.37–8.79; TT vs. C). This association was maintained in the analysis of the SNP’s influence on PFS since patients carrying the rs6068816-TT genotype had a higher risk of progression than those with the C allele (*p* = 0.0048; HR = 8.77; 95% CI = 1.94–39.7; TT vs. C) [10]. Likewise, Kong et al., in a study of 542 Asian patients (from China) with NSCLC, obtained a trend between this SNP and OS in the multivariate Cox regression model, where carriers of the CT genotype showed a tendency toward a higher risk of death than carriers of the CC genotype (*p* = 0.072; HR = 1.13; 95% CI = 0.86–1.49; CT vs. CC) [24]. In the case of the rs4809957 polymorphism, no statistically significant associations are reported for OS [10,26] or for PFS [10] in patients with NSCLC. 

#### 3.3.4. *GC*: Vitamin D Binding Protein (Group-Specific Component)

The rs7041 SNP located in the *GC* gene was studied in two of the articles included in this review [10,26]. The results of the univariate Cox regression model show that in one cohort of 48 Caucasian patients (from Spain) with resected NSCLC, carriers of the GG genotype presented a higher risk of death than those carrying the T allele (*p* = 0.0242; HR = 2.72; 95% CI = 1.14–6.47; GG vs. T). This association was clearer in the analysis of the influence of this SNP on PFS, where in the multivariate Cox regression model, those carrying the rs7041-GG genotype showed a higher risk of progression than those carrying the T allele (*p* = 0.044; HR = 2.26; 95% CI = 1.02–5.02; GG vs. T) [10]. On the other hand, Kong et al. did not observe a significant association between rs7041 and OS [26].

#### 3.3.5. *CYP2R1*: Cytochrome P450 Family 2 Subfamily R Member 1

Two studies included in this review analyzed the relationship between the rs10741657 polymorphism in the *CYP2R1* gene and OS and PFS [10,26]. Kong et al. report that in 542 Asian patients (from China) with NSCLC, in the multivariate Cox regression model, carriers of the AG and AA genotypes displayed a lower risk of death than those with the GG genotype (*p* = 0.033; HR = 0.79; 95% CI = 0.61–1.03; AG vs. GG, and *p* = 0.033; HR = 0.69; 95% CI = 0.46–0.97; AA vs. GG, respectively). In addition, in the subgroup analysis, they found that in 270 NSCLC patients aged over 60, those carrying the rs10741657-A allele had a lower risk of death than those with the GG genotype (*p* = 0.014; HR = 0.71; 95% CI = 0.51–0.99; A vs. GG). Finally, this association was also observed in the subgroup of 246 patients with NSCLC who did not receive chemotherapy. Patients with the rs10741657-A allele showed a lower risk of death than those carrying the GG genotype (*p* = 0.002; HR = 0.65; 95% CI = 0.45–0.95; A vs. GG) [26]. On the other hand, Pineda et al. did not observe any significant association between the rs10741657 SNP and OS or PFS [10]. 

### 3.4. Quality Assessment

The quality score assigned to each study included in the review is shown in Supplementary Table S2. The range of quality percentages obtained was 33.33–72.22%. Two studies fully reported the laboratory methods used (33.3%), while four studies were considered incomplete, as they did not indicate information on the DNA storage conditions or the genotyping platforms used (66.6%). Most of the studies included did not mention call rates and error rates (83.3%). Only one study took account of call rates (16.6%). No study reported the center at which the genotyping was performed (100%). All the studies mentioned the equipment with which the genotyping was done. However, they did not mention whether the genotyping was performed in one batch or in smaller batches (100%). All the studies reported both the number of samples to be genotyped and those that were finally genotyped successfully (100%). Three studies carried out stratified analyses (50%). Four studies performed haplotype inference (66.6%). Three studies mentioned the consideration of HWE in the analysis (50%). All the studies indicated whether their research involved reporting new associations, replicating previous studies, or both (100%).

## 4. Discussion

This is the first systematic review to have made a specific compilation of all the studies that have so far evaluated the influence of genetic polymorphisms in the main genes involved in the vitamin D metabolic pathway on OS and PFS in patients with NSCLC. The *VDR, CYP27B1, CYP24A1*, *GC,* and *CYP2R1* genes have proved to be the most significant [43,44,45,46,47,48]. These genes are the ones responsible for synthesizing the enzymes that mediate the first and second hydroxylation to activate vitamin D, the transport of vitamin D in its various isoforms, elimination, and binding to the vitamin D receptor, triggering the VDREs [43,44,45,46,47,49]. It is therefore presumed that SNPs in these genes may influence their functionality or expression and thereby affect the biological activity of vitamin D. Consequently, these variations could be considered as predictive biomarkers of the outcome of the disease. 

The *VDR* gene (also known as NR1I1) is located on chromosome 12 in the 12q13.11 region. It encodes a ligand-dependent transcription factor belonging to the nuclear receptor (NR) superfamily [50,51,52,53]. The SNPs in this gene have been extensively studied, and BsmI (rs1544410), TaqI (rs731236), ApaI (rs7975232), FokI (rs2228570), and Cdx-2 (rs11568820) are those that have proved to be most important. Two of the studies included in this review reveal that the BsmI (rs1544410) SNP was associated with a higher risk of death in patients with NSCLC [11,25]. These results are in line with a multiethnic meta-analysis conducted with 9926 cases in 10 studies of various types of cancer (prostate, lung, head and neck, colorectal, skin, and glioma), which reveals that the TT/TC genotypes for the *VDR* BsmI (rs1544410) polymorphism were associated with worse OS (HR = 1.40; 95% CI = 1.05–1.75; I2 = 0.85) [46]. For the Cdx-2 (rs11568820) polymorphism, we have found contradictory results. On the one hand, a study reports that in 108 Caucasian patients (from the United States) with early-stage NSCLC and the squamous cell histological subtype, those carrying the A allele showed better OS and better PFS [29], whereas another study of 48 Caucasian patients (in Spain) with resected NSCLC reported that carriers of the AA genotype had worse survival [10]. However, when the results were compared with a multiethnic meta-analysis conducted with 9921 cases in nine studies of various types of cancer (breast, prostate, lung, colorectal, skin, and glioma), no statistically significant association was found between this SNP and OS (HR = 1.00; 95% CI = 0.84–1.17; I2 = 0) [44]. As for the FokI (rs2228570) SNP, a study conducted on 294 Caucasian patients (from the United States) reveals that the T allele was associated with a higher risk of death in advanced NSCLC [25]. This result is in line with a meta-analysis conducted on Caucasian populations (from the United States) with 667 lung cancer cases in two studies, where the TT genotype was associated with worse survival (HR = 1.29; 95% CI = 1.00–1.57; I2 = 0) [44]. Similarly, a constant, though not significant, association was found between the TT genotype and OS in all cancers (breast, prostate, lung, colorectal, head and neck, skin, glioma, and ovarian; 11,334 cases in 12 studies) (HR = 1.26; 95% CI = 0.96–1.56; I2 = 0.83) [44]. Two of the studies, conducted on 755 Asian patients (from China) and 146 Caucasian patients (from Spain) found that the AA genotype for ApaI (rs7975232) was associated with a higher risk of death and progression in advanced NSCLC [10,28]. These results are in line with those reported in a multiethnic meta-analysis with 1588 cases in 3 studies of 3 types of cancer (breast, prostate, and lung), where carriers of the AA genotype for ApaI (rs7975232) had significantly worse survival (HR = 1.29; 95% CI = 1.02–1.56; I2 = 0) [44]. Finally, for the TaqI (rs731236) polymorphism, two of the studies included, conducted on 586 Asian patients (from China) and 146 Caucasian patients (from Spain), revealed that the GG genotype was associated with a higher risk of death and in one study, it was associated with a higher risk of progression in non-resected NSCLC [10,27]. These results were not maintained in a multiethnic meta-analysis with 8583 cases in 9 studies in various types of cancer (breast, prostate, colorectal, lung, head and neck, skin, and glioma) where no statistically significant results were found in relation to survival (HR = 1.34; 95% CI = 0.89–1.79; I2 = 0.86) [44]. These results may be explained, on the one hand, by the fact that vitamin D-activating enzymes and *VDR* are present in many tissues, including lung tissue [51,54,55,56]. It is estimated, moreover, that *VDR* and its ligand regulate 1–3% of all gene expression [57,58], including the genes involved in calcium metabolism, cell growth, anti-proliferation, differentiation, apoptosis, and adaptive/innate immune responses [50,51,52,59]. On the other hand, it has been reported that increased expression of *VDR* in lung cancer is associated with better survival [60,61]. This may be related to a lower proliferative state and the G1-phase arrest of tumor cells with high expression of VDR [60,61]. 

The *CYP27B1* gene codes for 1-alpha-hydroxylase, the only enzyme capable of converting vitamin D into its active form. The gene is located at 12q14.1 in the long arm of chromosome 12 [62,63]. This systematic review included the rs10877012, rs4646536, rs3782130, and rs703842 SNPs. It revealed that the A allele of rs4646536 was associated with a higher risk of progression. These results are in line with a study conducted on an Asian population (China) with 528 colon cancer patients, where it was found that for rs4646536, patients carrying the AA genotype had a worse prognosis than those with the G allele (plog-rank = 0.01) [64]. Likewise, the rs10877012 and rs3782130 SNPs were related to a higher risk of progression in 146 Caucasian patients (from Spain) with non-resected NSCLC [10]. However, this association was not maintained in the multivariate Cox regression. The information reported to date in the scientific literature on the association of these SNPs with survival in cancer is scarce. Research has focused on determining the influence of polymorphisms on gene expression. A study conducted on an Asian population (from China) with 153 tumor samples (NSCLC) found that high *CYP27B1* expression was associated with better OS (*p* = 0.018). It also found, in turn, that changes in gene expression can be due to SNPs. Specifically, it found that differences in expression were statistically significant in the rs3782130 polymorphism (*p* = 0.028) [45]. Moreover, it has been shown that expression of the *CYP24A1*, *CYP27B1*, and *VDR* genes in lung cancer is affected by tumor differentiation and characterization. When the tumor is poorly differentiated, expression of *CYP24A1* is increased and that of *CYP27B1* is reduced [56,65].

Vitamin D metabolism is catalyzed by the 24-hydroxylase enzyme, which is responsible for carrying out a series of hydroxylations to convert calcitriol into calcitroic acid, a molecule with greater polarity to facilitate its elimination via the kidneys [30,65,66,67]. The 24-hydroxylase enzyme is encoded by the *CYP24A1* gene, located on chromosome 20, specifically in the 20q13.2 region [56,65]. One of the studies included in this review, conducted on 146 Caucasian patients (from Spain), reports an association between carriers of the TT genotype for the *CYP24A1* rs6068816 SNP and increased risk of death and progression in patients with non-resected NSCLC [10]. These results are in line with a multiethnic meta-analysis with 1419 cases in 5 studies in 4 types of cancer (head and neck, lung, breast, and colorectal), which evaluated the impact of 5 SNPs with some role in cancer survival (rs2296241, rs6068816, rs2762934, rs4809957, rs6013897) on survival, finding that the polymorphisms of *CYP24A1* were positively associated with a worse prognosis (HR = 1.42; 95% CI = 1.16–1.68; I2 = 0.0) [66]. Furthermore, there are studies that relate increased expression of *CYP24A1* to a worse prognosis, since it has been observed that when the tumor is poorly differentiated, gene expression is greater [56,65]. Although the information on the influence of SNPs on tumor expression is unclear, it is known that synonymous polymorphisms such as rs6068816, which does not alter the amino acid sequence of *CYP24A1*, may influence intron splicing. This affects the patterns or efficiency of mRNA splicing, which in turn has an impact on biological activities [45,68]. This information was observed in a multiethnic meta-analysis with 761 cases in 4 studies in two types of cancer (lung and colorectal), where increased CYP24A1 protein expression correlated with worse survival (HR = 1.14; 95% CI = 1.02–1.26; I2 = 36) [69]. Likewise, the influence of *CYP24A1* mRNA expression on survival in 3 types of cancer (lung, esophageal, and breast) was assessed in a multiethnic meta-analysis with 1479 cases in 4 studies; it revealed that elevated mRNA expression was associated with worse prognosis (HR = 1.24; 95% CI = 1.01–1.46; I2 = 38.1) [69]. The results observed in the various studies may be related to the abrogation of the antiproliferative effects of calcitriol with increased *CYP24A1* expression in lung tumor tissue [67]. 

The *GC* gene, located in the 4q13.3 region of chromosome 4, belongs to the albumin superfamily of binding proteins (albumin, alpha-fetoprotein, alpha-albumin/afamin). This gene is characterized by synthesizing the vitamin D binding protein (VDBP), responsible for the blood transport of vitamin D metabolites to the targets of action [70,71,72]. One of the studies included in this review, conducted on 48 Caucasian patients (from Spain), showed that carriers of the GG genotype of *GC* rs7041 were associated with a higher risk of death and progression [10]. There is limited evidence on the influence of the *GC* rs7041 polymorphism on survival in cancer. The available studies have focused on gene expression and circulating VDBP levels. A study of 148 Caucasian lung cancer patients (from the UK) revealed that low serum VDBP levels predicted lung cancer-specific death (*p* = 0.04) since VDBP was poorly expressed in tumor tissue cells [73]. On the other hand, a study conducted on 381 Asian participants (from Jordan) found that in patients with chronic diseases, the *GC* rs7041-TT genotype was associated with higher 25(OH)D levels (*p* = 0.007) [74]. A study conducted on 4038 Caucasian patients (from the USA) diagnosed with 11 malignancies found that higher concentrations of 25(OH)D were associated with greater survival in lung cancer (*p* = 0.03; HR = 0.63; 95% CI = 0.44–0.90) and a trend toward greater overall cancer survival (*p* = 0.05; HR = 0.83; 95% CI = 0.70–0.98). These associations were limited to cases that expressed the Gc2 isoform (rs7041-T/rs4588-T) [75].

The 25-hydroxylase or CYP2R1 enzyme is encoded by the gene of the same name (*CYP2R1*), located on chromosome 11, in the 11p15.2 region. This enzyme is responsible for synthesizing calcidiol or 25(OH)D from its precursors, ergocalciferol (vitamin D2) and cholecalciferol (vitamin D3) [45,46,76]. Two studies were found that evaluated the effect of *CYP2R1* rs10741657 polymorphism on survival in NSCLC. First, Pineda et al. in the study performed on 194 Caucasian patients (from Spain), found a strong tendency for higher risk of death in carriers of the rs10741657-AA genotype in the univariate Cox regression model [10]. On the other hand, a study of 542 Asian patients (from China) found that the A allele of *CYP2R1* rs10741657 was associated with a better prognosis in both stage I-IV NSCLC and subgroup analysis [26]. Differences between these findings may be mainly due to the ethnicity of the populations studied, the sample size, the total follow-up time of the study, and the confounding variables included in each of the investigations [26]. Nevertheless, these results are in line with those reported in a Mendelian randomization study with 95,766 Caucasian participants (from Denmark), which revealed that low serum vitamin D levels increased cancer mortality (HR = 1.12; 95% CI = 1.03–1.22) and all-cause mortality (HR = 1.19; 95% CI = 1.14–1.25). It also determined that low vitamin D levels could be due to polymorphisms in *CYP2R1* and *DHCR7*. In particular, carriers of the rs10741657 GG genotype were associated with lower 25-hydroxyvitamin D levels [54]. Other authors have reported that the transition mutation in exon 2 of the *CYP2R1* gene leads to the substitution of proline for leucine at amino acid 99 in the CYP2R1 protein and suppresses the enzyme activity of vitamin D 25-hydroxylase, indicating that genetic variation in this gene may affect 25(OH)D synthesis [77,78,79]. 

The differences observed between results regarding the impact of certain SNPs on OS or PFS may be mainly due to the high methodological heterogeneity between the studies included, in terms of ethnicity, sample size, and clinical data collection, for example, as well as in the performance of subgroup analyses, where the power of the associations is altered by the small sample size.

This review has some limitations, including the following: (I) The inclusion of studies that analyzed the influence of genetic polymorphisms in the vitamin D metabolic pathway on patients with NSCLC, which restricts the possible number of results and prevents them from being extrapolated to other malignancies. (II) Moreover, only the influence of SNPs on OS and PFS was examined, excluding the possible effect of these genetic variants on the risk of developing the disease. (III) Owing to the scarcity of results found and the reporting of results by subgroups, it was not possible to perform a meta-analysis to observe variations in the level of association of the genotypes studied with the disease prognosis. (IV) The studies included were in the low to moderate methodological quality range according to the STREGA statement criteria, and therefore the interpretations of the findings of this review must be treated with caution. Despite these limitations, this study includes an exhaustive compilation of scientific reports that have investigated the association between the main genetic variants related to the vitamin D metabolic pathway and the prognosis of NSCLC. The information endorses the importance of the biological functions of vitamin D and its influence on cancer. 

## 5. Guidelines for Future Research 

The information provided in this systematic review is intended to prepare the ground for conducting randomized clinical trials, with greater statistical power, to evaluate the clinical application of these genetic polymorphisms as prognostic biomarkers of disease, which, together with other biomarkers, such as protein expression, genomics, molecular markers, and clinical biomarkers, may provide a more accurate assessment of prognosis and a better treatment strategy for each patient on an individualized basis.

Additionally, future research could focus on designing strategies that adapt to different changes that may arise in the vitamin D metabolic pathway, including evaluating the implementation of vitamin D in its active form as an adjuvant chemotherapeutic agent.

## 6. Conclusions

The results obtained in this systematic review suggest that the rs10741657, rs4646536, rs7041, rs6068816, BsmI, Cdx-2, FokI, ApaI, and TaqI SNPs in genes related to synthesis (*CYP2R1*, *CYP27B1*), transport (*GC*), metabolism (*CYP24A1*), and binding to the vitamin D receptor (*VDR*) could be considered predictive biomarkers of OS and PFS in patients with NSCLC. However, the evidence remains scarce for each of the polymorphisms reviewed and the studies display methodological differences and small sample sizes, making it impossible to reach firmer conclusions.

## Figures and Tables

**Table 1:** Search strategy.

Non-small-cell lung cancer OR lung cancer OR NSCLC
AND
vitamin D receptor OR VDR OR rs1544410 OR BsmI OR rs2228570 OR FokI OR rs7975232 OR ApaI OR rs11568820 OR Cdx-2 OR rs731236 OR TaqI OR CYP27B1 OR 1-a-hydroxylase OR rs10877012 OR rs4646536 OR rs3782130 OR rs703842 OR CYP2R1 OR 25-hydroxylase OR rs10741657 OR CYP24A1 OR 24-hydroxylase OR rs6068816 OR rs4809957 OR GC OR VDBP OR rs7041 OR vitamin D binding protein.
AND
Survival OR progression-free survival OR prognosis OR mortality OR death.

We included the additional term vitamin D.

**Table 2:** Characteristics of the included PGx studies.

First Author (year)	Ethnicity	Study Design	Sample Size (Deaths/ Total)	NSCLC Stage	Median Follow-Up	*VDR*					*CYP27B1*				*CYP24A1*		*GC*	*CYP2R1*	Outcome		PMID
						BmsI	Cdx-2	FokI	ApaI	TaqI	rs10877012	rs4646536	rs3782130	rs703842	rs6068816	rs4809957	rs7041	rs10741657	OS	PFS	
Zhou et al. (2006) [29]	Caucasians (USA)	Cohort	186/373	IA-IIB	71 months	X	X	X											X	X	17119052
Heist et al. (2008) [25]	Caucasians (USA)	Cohort	233/294	III-IV	42 months	X	X	X											X		18936471
Liu et al. (2011) [27]	Asiatic (China)	Cohort	311/568	I-IV	19 months	X	X		X	X									X		23467735
Xiong et al. (2013) [28]	Asiatic (China)	Cohort	NA/755	III-IV	NA	X		X	X	X									X	X	23522953
Kong et al. (2020) [26]	Asiatic (China)	Cohort	278/542	I-IV	80 months ^a^						X	X	X	X	X	X	X	X	X		31625015
Pineda et al. (2021) [10]	Caucasians (Spain)	Cohort	154/194	I-IV	204 months ^a^	X	X	X	X	X	X	X	X		X	X	X	X	X	X	34836039

^a^: Total follow-up; NA: Not available; OS: Overall survival; PFS: Progression-free survival.

**Table 3:** SNPs associated with Overall Survival and Progression-free Survival in NSCLC patients.

dbSNP ID	SNP Position	Frequency (ALFA)	Sample Size	Overall Survival (OS)				Progression-Free Survival (PFS)				PMID
				log Rank *p*	Ref.	HR (95% CI)	Comments	log Rank *p*	Ref.	HR (95% CI)	Comments	
Gene *VDR* (12q13.11)												
rs1544410 (BsmI)	Intron 8, C > T	T = 0.388066 (85636/220674)	373 early-stage NSCLC	0.31	CC	0.83 (0.59–1.16) CT 1.35 (0.90–2.03) TT	Multivariate Cox regression	>0.05				17119052 [29]
			180 early-stage Adenocarcinoma	0.30	CC	0.88 (0.52–1.48) CT 1.52 (0.81–2.83) TT	Multivariate Cox regression	>0.05				17119052 [29]
			108 early-stage Squamous	0.83	CC	0.59 (0.33–1.05) CT 1.18 (0.62–2.23) TT	Multivariate Cox regression	>0.05				17119052 [29]
			294 Advanced NSCLC	0.61	CC	0.89 (0.66–1.19) CT 0.93 (0.64–1.35) TT	Multivariate Cox regression					18936471 [25]
			562 NSCLC	0.008 0.004	CC CC	1.55 (1.09–2.21) CT 4.33 (1.34–14.0) TT 1.64 (1.16–2.31) T	Multivariate Cox regression					23467735 [27]
			755 Advanced NSCLC	>0.05				>0.05				23522953 [28]
			194 NSCLC	0.500				0.900				34836039 [10]
			48 resected NSCLC	0.600				0.700				34836039 [10]
			146 non-resected NSCLC	0.0073	C	2.08 (1.22–3.56) TT	Univariate Cox Model	0.500				34836039 [10]
rs11568820 (Cdx-2)	Intron 1, G > A	A = 0.28140 (14569/51774)	373 early-stage NSCLC	0.37	GG	0.84 (0.62–1.14) AG 0.92 (0.50–1.68) AA	Multivariate Cox regression	>0.05				17119052 [29]
			180 early-stage Adenocarcinoma	0.33	GG	1.02 (0.64–1.62) AG 1.71 (0.81–3.60) AA	Multivariate Cox regression	>0.05				17119052 [29]
			108 early-stage Squamous	0.05 0.04	GG GG	0.55 (0.32–0.95) AG 0.69 (0.16–2.96) AA 0.56 (0.33–0.95) A	Multivariate Cox regression	0.03	GG	0.57 (0.34–0.94) A	Multivariate Cox regression	17119052 [29]
			294 Advanced NSCLC	0.59	GG	0.85 (0.63–1.15) GA 1.02 (0.64–1.63) AA	Multivariate Cox regression					18936471 [25]
			586 NSCLC	0.773 0.957 0.470		Additive Dominant Recessive	Log-rank P					23467735 [27]
			194 NSCLC	0.400				0.600				34836039 [10]
			48 resected NSCLC	0.0129	G	7.43 (1.53–36.15) AA	Multivariate Cox regression	0.055	G	4.34 (0.97–19.5) AA	Univariate Cox Model	34836039 [10]
			146 non-resected NSCLC	0.700				0.400				34836039 [10]
rs2228570 (FokI)	Exon 2, C > T	T = 0.388743 (91849/236272)	373 early-stage NSCLC	0.93	CC	0.84 (0.61–1.16) CT 1.13 (0.74–1.74) TT	Multivariate Cox regression	>0.05				17119052 [29]
			180 early-stage Adenocarcinoma	0.40	CC	1.13 (0.67–1.88) CT 1.31 (0.70–2.46) TT	Multivariate Cox regression	>0.05				17119052 [29]
			108 early-stage Squamous	0.64	CC	0.75 (0.44–1.28) CT 0.98 (0.47–2.03) TT	Multivariate Cox regression	>0.05				17119052 [29]
			294 Advanced NSCLC	0.04	CC	1.32 (0.98–1.77) CT 1.41 (0.96–2.07) TT	Multivariate Cox regression					18936471 [25]
			755 Advanced NSCLC	>0.05				>0.05				23522953 [28]
			194 NSCLC	0.600				0.600				34836039 [10]
			48 resected NSCLC	1.000				0.400				34836039 [10]
			146 non-resected NSCLC	0.700				0.400				34836039 [10]
rs7975232 (ApaI)	Intron 8, C > A	C = 0.44552 (17435/39134)	586 NSCLC	NR			Removed (ApaI was not in HWE)					23467735 [27]
			755 Advanced NSCLC	<0.001	CC	2.84 (2.63–3.94) AA	Multivariate Cox regression	0.053	CC	1.43 (0.99–2.78) AA	Multivariate Cox regression	23522953 [28]
			194 NSCLC	0.400				0.600				34836039 [10]
			48 resected NSCLC	1.000				1.000				34836039 [10]
			146 non-resected NSCLC	0.0068	C	1.73 (1.16–2.58) AA	Univariate Cox Model	0.0002	C	3.08 (1.71–5.54) AA	Multivariate Cox regression	34836039 [10]
rs731236 (TaqI)	Exon 9, A > G	G = 0.387180 (74890/193424)	586 NSCLC	0.027 0.016	AA AA	1.41 (1.00–1.99) AG 4.26 (1.32–13.8) GG 1.49 (1.07–2.08) G	Multivariate Cox regression					23467735 [27]
			755 Advanced NSCLC	>0.05				>0.05				23522953 [28]
			194 NSCLC	0.200				0.900				34836039 [10]
			48 resected NSCLC	0.700				0.500				34836039 [10]
			146 non-resected NSCLC	0.0005	A	2.71 (1.55–4.75) GG	Multivariate Cox regression	0.0463	A	1.74 (1.01–2.99) GG	Multivariate Cox regression	34836039 [10]
*CYP27B1* (12q14.1)												
rs10877012	5′UTR, G > T	T = 0.292364 (46918/160478)	542 NSCLC	0.695	TT	1.28 (0.69–1.97) TG 1.39 (0.38–1.85) GG	Multivariate Cox regression					31625015 [26]
			194 NSCLC	0.098	TT	1.826 (0.89–3.73) G	Univariate Cox Model	0.400				34836039 [10]
			48 resected NSCLC	0.0827	T	2.42 (0.89–6.58) GG	Univariate Cox Model	0.400				34836039 [10]
			146 non-resected NSCLC	0.200				0.044	T	2.05 (1.02–4.14) GG	Univariate Cox Model	34836039 [10]
rs4646536	Intron 6, A > G	G = 0.32704 (27483/84036)	542 NSCLC	0.625	GG	1.42 (0.73–2.74) GA 1.43 (0.68–3.04) AA	Multivariate Cox regression					31625015 [26]
			194 NSCLC	0.056	GG	2.01 (0.98–4.14) A	Multivariate Cox regression	0.023	GG	2.11 (1.11–4.04) A	Multivariate Cox regression	34836039 [10]
			48 resected NSCLC	0.0676	G	2.54 (0.93–6.89) AA	Univariate Cox Model	0.300				34836039 [10]
			146 non-resected NSCLC	0.200				0.004	G	8.77 (1.94–39.7) AA	Multivariate Cox regression	34836039 [10]
rs3782130	Promotor 5′, G > C	C = 0.18262 (2560/14018)	542 NSCLC	0.263	CC	0.63 (0.22–1.77) CG 1.16 (0.33–4.18) GG	Multivariate Cox regression					31625015 [26]
			194 NSCLC	0.200				0.400				34836039 [10]
			48 resected NSCLC	0.0827	C	2.42 (0.89–6.59) GG	Univariate Cox Model	0.400				34836039 [10]
			146 non-resected NSCLC	0.200				0.045	C	2.05 (1.01–4.13) GG	Univariate Cox Model	34836039 [10]
rs703842	3′UTR, T > C	C = 0.326395 (80258/245892)	542 NSCLC	0.627	CC	1.27 (0.67–3.25) CT 1.16 (0.45–2.78) TT	Multivariate Cox regression					31625015 [26]
*CYP24A1* (20q13.2)												
rs6068816	Exon 6, C > T	T = 0.108153 (33297/307870)	542 NSCLC	0.072	CC	1.13 (0.86–1.49) CT 0.76 (0.49–1.19) TT	Multivariate Cox regression					31625015 [26]
			194 NSCLC	0.900				1.000				34836039 [10]
			48 resected NSCLC	0.117	T	4.99 (0.67–37.2) CC	Univariate Cox Model	0.0359	T	8.49 (1.15–62.67) CC	Univariate Cox Model ^a^	34836039 [10]
			146 non-resected NSCLC	0.0089	C	3.47 (1.37–8.79) TT	Multivariate Cox regression	0.0048	C	8.77 (1.94–39.7) TT	Multivariate Cox regression	34836039 [10]
rs4809957	3′UTR, A > G	G = 0.232972 (62226/267096)	542 NSCLC	0.790	GG	0.97 (0.74–1.26) GA 0.92 (0.58–1.45) AA	Multivariate Cox regression					31625015 [26]
			194 NSCLC	0.300				0.089	G	2.03 (0.89–4.59)	Univariate Cox Model	34836039 [10]
			48 resected NSCLC	0.700				0.700				34836039 [10]
			146 non-resected NSCLC	0.700				0.900				34836039 [10]
*GC* (4q13.3)												
rs7041	Exon 11, T > G	T = 0.457674 (154076 /336650)	542 NSCLC	0.693	TT	0.82 (0.64–1.07) TG 1.13 (0.67–1.92) GG	Multivariate Cox regression					31625015 [26]
			194 NSCLC	0.300				0.300				34836039 [10]
			48 resected NSCLC	0.0242	T	2.72 (1.14–6.47) GG	Univariate Cox Model	0.044	T	2.26 (1.02–5.02) GG	Multivariate Cox regression	34836039 [10]
			146 non-resected NSCLC	0.700				0.400				34836039 [10]
*CYP2R1* (11p15.2)												
rs10741657	5′UTR, A > G	A = 0.379194 (73776/194560)	542 NSCLC	0.033	GG	0.79 (0.61–1.03) GA 0.69 (0.46–0.97) AA	Multivariate Cox regression					31625015 [26]
			270 NSCLC (Age group >60)	0.014	GG	0.71 (0.51–0.99) A	Multivariate Cox regression					31625015 [26]
			246 NSCLC (Chemotherapy: No)	0.002	GG	0.65 (0.45–0.95) A	Multivariate Cox regression					31625015 [26]
			194 NSCLC	0.0525	G	1.58 (0.99–2.52) AA	Univariate Cox Model	0.300				34836039 [10]
			48 resected NSCLC	0.800				0.300				34836039 [10]
			146 non-resected NSCLC	0.700				1.000				34836039 [10]

NR: not reported; SNP: Single nucleotide polymorphisms; ALFA: Allele Frequency Aggregator; Ref.: Reference allele; HR: Hazard ratio; CI: Confidence interval; HWE: Hardy–Weinberg equilibrium; ^a^: This SNP was excluded from the analysis; Shade means the value is significant.

## Data Availability

Not applicable, due to this being a systematic review. All data are available in primary studies.

## Supplementary Materials

The following supporting information can be downloaded at https://www.mdpi.com/article/10.3390/nu15061525/s1, Table S1: PRISMA 2020 checklist; Table S2: Reporting quality of the included pharmacogenetic studies.

## Abbreviations

CI: confidence interval; HR: hazard ratio; HWE: Hardy–Weinberg equilibrium; LD: linkage disequilibrium; NR: nuclear receptor; NSCLC: non-small cell lung cancer; OS: overall survival; PGx: pharmacogenetics; PFS: progression-free survival; PRISMA: Preferred Reporting Items for Systematic Reviews and Meta-analyses; SNP: single-nucleotide polymorphism; STREGA: Strengthening the Reporting of Genetic Association Studies; VDBP: vitamin D binding protein; VDRE: vitamin D response element; WHO: World Health Organization.
