# Supplementary material for: Polymorphisms in VDR, CYP27B1, CYP2R1, GC and CYP24A1 Genes as Biomarkers of Survival in Non-Small Cell Lung Cancer: A Systematic Review
Source: Nutrients. 2023 Mar 21;15(6):1525. doi: 10.3390/nu15061525 (PMC10057500; doi:10.3390/nu15061525)
Supplement: Supplementary file 1 [file nutrients-15-01525-s001.zip › Table S2.pdf]

Table S2. Reporting quality of the included pharmacogenetic studies

Study	Item 1	Item 2	Item 3	Item 4	Item 5	Item 6	Item 7	Item 8	Item 9	Score (%)
Zhou et al. (2006)	I	N	N	I	Y	Y	Y	Y	Y	6 (66.6)
Heist et al. (2008)	Y	N	N	I	Y	N	Y	N	Y	4.5 (50)
Liu et al. (2011)	I	I	N	I	Y	N	Y	Y	Y	5.5 (61.1)
Xiong et al. (2013)	I	N	N	I	Y	N	N	N	Y	3 (33.3)
Kong et al. (2020)	I	N	N	I	Y	Y	N	N	Y	4 (44.4)
Pineda et al. (2021)	Y	N	N	I	Y	Y	Y	Y	Y	6.5 (72.2)
Item										
Y %	2 (33.3)	0 (0.0)	0 (0.0)	0 (0.0)	6 (100)	3 (50.0)	4 (66.6)	3 (50.0)	6 (100)	
I %	4 (66.6)	1 (16.6)	0 (0.0)	6 (100)	0 (0.0)	0 (0.0)	0 (0.0)	0 (0.0)	0 (0.0)	
N %	0 (0.00)	5 (83.3)	6 (100)	0 (0.0)	0 (0.0)	3 (50.0)	2 (33.3)	3 (50.0)	0 (0.0)	

I, incomplete. N, no. Y, yes.

Item description: Item 1. Describe laboratory methods, including source and storage of DNA, genotyping methods and platforms; Item 2: Describe error rate and call rate; Item 3: State the laboratory/center at which genotyping was performed; Item 4: Provide a hint on whether the genotypes were assigned in one single batch or a few smaller batches; Item 5: Report number of individuals in whom genotyping was attempted to and how many of these samples were successfully genotyped; Item 6: Describe methods used to assess/address population stratification; Item 7: Describe any methods used for inferring genotypes or haplotypes; Item 8: Stated whether Hardy–Weinberg equilibrium was considered; Item 9: State if the study is the first report to report such genetic association, a replication or both.
